# Famine-Dropsy as a Food-Deficiency Disease
*A paper read at the meeting of the Bristol Medico-Chirurgical Society on May 12th, 1920.


**Published:** 1920-09

**Authors:** J. A. Nixon

**Affiliations:** Physician to the Bristol Royal Infirmary; Consulting Physician to the Bristol Guardians at Southmead Hospital; formerly Consulting Physician to the British Armies in France and to the Army of the Rhine


The Bristol
Medico-Chirurgical Journal.

*" Scire est nescire, nisi id me
Scire alius sciret."*

SEPTEMBER, 1920.

### FAMINE-DROPSY AS A FOOD-DEFICIENCY DISEASE.*

BY

J. A. Nixon, C.M.G., M.D., F.R.C.P.,

*Physician to the Bristol Royal Infirmary; Consulting Physician to the Bristol Guardians at Southmead Hospital; formerly Consulting Physician to the British Armies in France and to the Army of the Rhine.*

Under the names of War Œdema and Hunger Œdema an old form of dropsy was described during the Great War as a new disease. Dropsy had been recognised as the result of under-feeding and famine since the time when the soldiers of the French Army before Naples in 1528 had crawled about with pallid visages, swelled legs, and bloated bellies.[1] It had been recorded in besieged towns, in ships making long cruises and in famine-stricken countries. Great confusion had existed until recent times between famine-dropsy, scurvy and beriberi. Cornish described the condition with great accuracy as occurring in the Madras jails in 1864,[2] and again in the Indian famine of 1877–8.[3]

* A paper read at the meeting of the Bristol Medico-Chirurgical Society on May 12th, 1920.

During 1915 outbreaks occurred in German prison camps in association with various epidemic diseases, such as relapsing fever, dysentery and typhus. At the same time it was observed among the famine-stricken populations in Galicia and Poland. From a recently-published report it is evident that the earliest appearance of famine-dropsy during the War was at Lille in October, 1914.[4] The unhappy town was in German occupation, and the inhabitants were suddenly stripped of everything, so that many of them had nothing to eat except potatoes. This diet produced many cases of a general anasarca unaccompanied by albuminuria. My own experience of the disease was derived from seeing a number of slight and a few severe cases amongst released prisoners of war who were making their way back to France immediately after the Armistice, or who were found in the hospitals of the re-occupied portions of France and Belgium during the advance of the Fourth Army.

In this paper, necessarily much compressed, I have tried to give a short description of famine-dropsy. A fuller and comprehensive discussion of the disease in all its aspects will shortly be published in the medical history of the war.

The disease may be defined as a form of dropsy associated with bradycardia, polyuria and asthenia, which occurs in persons subjected to prolonged underfeeding. It is unattended by albuminuria, cardiac dilatation or neuritis. It affects more particularly men who are called upon to perform hard physical work whilst their daily food ration contains from 800 to 1200 calories. These calories are as a rule embodied in a largely fluid diet which comprises 15 per cent. or more of indigestible cellulose with very little fat and a maximum daily allowance of 50 grammes of protein.

The onset is gradual, with a few days' malaise, during which the patient complains of little beyond great lassitude, physical weakness, headache, and a noticeable increase in the quantity of urine passed. Then the feet and ankles begin to swell. The œdema commences in the dependent parts and spreads to the limbs and trunk. Sometimes it involves the whole body, including even the face and hands. Ascites, hydrothorax and hydropericardium are of frequent occurrence. The skin assumes a pale yellow tint and muscular wasting may be extreme. There is usually very marked apathy.

At the first onset of œdema a remarkable polyuria is invariably observed. It takes the form of nocturnal frequency sufficient to interfere seriously with sleep. In the more apathetic patients enuresis is common.

When with appropriate treatment the dropsy disappears the emaciation becomes obvious. This emaciation is an integral part of the disease, and is always very great. It corresponds with the total loss of subcutaneous and other fat revealed by autopsy, and is a main point of distinction between famine-dropsy and that due to renal or cardiac disease. The heart's action becomes very slow. Extreme bradycardia without irregularity occurs in almost all cases; the rate is usually between 40 and 50 per minute, but a rate of 26 has been recorded. This bradycardia is of sinus origin. The heart sounds are faint and muffled. Sometimes a soft hæmic bruit is heard over all the valvular areas. The pulse is small and feeble, the blood-pressure low (90–100 mm. Hg.). The lungs are as a rule normal, unless there is some complicating broncho-pneumonia or hydrothorax. Dyspnœa, cyanosis and signs of failing pulmonary circulation are conspicuously absent. The liver and spleen are not enlarged.

There are no changes referable to the nervous system such as characterise beriberi. Although scorbutic manifestations may be present, they are not part of the famine-dropsy, but rather of a co-existing scurvy. The same is true of the skin changes of pellagra, which do not appear in uncomplicated cases of famine-dropsy. The temperature is normal or subnormal. Fever if present depends upon some complication. Diarrhœa is a symptom so constantly present as to raise the question whether it may not play a part in causing the disease. There seems some relation between the indigestible residue of the food and the diarrhœa. But it is quite certain that famine-dropsy can occur in an outbreak in which diarrhœa is totally absent.

The renal functions seem to be normal. The changes in the urine are probably due to variations in the intake. Suppression of urine is never seen with true famine-dropsy; on the contrary, polyuria is the rule. The polyuria probably depends on polydipsia, with which it goes hand in hand. No albumin, casts or sugar are present. As regards mineral constituents, most observers agree that there is a marked increase in the chlorides, but this may be due to an increased intake: in prison camps men used to add large amounts of common salt to the thin soups to try to give some flavour to them. Calcium phosphates and magnesium show less variation from the normal.

The nitrogen balance is undisturbed. The excretion of urea is low, but improves with the addition of protein to the diet. Ammonia-nitrogen and kreatin are increased as in all forms of starvation with disintegration of the tissue-proteins. Amino-acid-nitrogen and kreatinin sometimes show a slight and variable increase. Urates are as a rule increased in the early stages. Acetone has occasionally been found in the urine.

In the blood hydræmia is constantly present, but does not correspond with the degree of œdema. The red-cell count may vary within wide limits. Usually there is a moderate degree of oligocythæmia (3 to 5 million). The hæmoglobin index is normal. In cases with less than three million erythrocytes cell-changes have been observed: basophilic granules with polychromatophilia, a slight poikilo- and aniso-cytosis and in a few cases normoblasts.

Leucopenia is generally present, the number of leucocytes commonly being between 2,000 and 8,000 per c.mm. Where the count rises above 8,000 some complication may be suspected. There is a relative decrease in neutrophiles and an increase in mononuclears, chiefly the large mononuclears, of which the proportion may reach 55 per cent. These cells frequently attain a very large size, and may contain numerous coarse basophile granules. Eosinophiles and basophiles are often increased.

There is a constant and definite fall in the total proteins of the blood-serum, which shows the same variations in its nitrogen constituents as the urine. The fat-content of the serum remains normal. The lipoid-phosphorus is greatly decreased, whilst the acid-phosphates are increased. The lecithin content of the erythrocytes is diminished and the cholesterin unchanged.

To sum up: there are no changes in the composition of the blood and urine which cannot be explained as the normal outcome of deficiencies and excess in the food-constituents.

*Pathogenesis.*—We meet with many difficulties, however, when we try to explain why one form of starvation should produce dropsy whilst another does not. Complete starvation leads to death in eight or ten days without the appearance of dropsy. Certain forms of inanition are, however, associated with œdema. We see this not infrequently in acute dysentery and in the last stages of phthisis. As Edgeworth observed in describing the death of five out of six infants in one family with general œdema following upon diarrhœa, "it is well known that dropsy, especially of the face and extremities, may occur in infants suffering from chronic diarrhœa without albuminuria." [5]

The essential conditions which lead to famine-dropsy pure and simple are well established, namely prolonged under-feeding with a largely fluid diet poor in calories, deficient in protein and fat. Climate plays some part; exposure and fatigue hasten the onset of dropsy. Previous illnesses, such as diarrhœa and dysentery, so constantly precede it, that at one period of the war it was suspected that the disease might be due to an intestinal infection. In the Great War it was seen that in a given community fed on the same ration more cases occurred in cold weather than in warm. But the Indian famine of 1877–8 (when Cornish first recognised famine-dropsy as a definite entity differing from beriberi and scurvy) demonstrated that famine-dropsy can occur in hot climates as well as cold.

The ingestion of fluid in large quantity is a contributing factor. The German ration for prisoners was given almost entirely in the form of soup, to which a large amount of salt was added. An excess of sodium chloride is not essential to the production of this form of dropsy, as I have seen it occur in a patient in hospital whose intake of salt was infinitesimal.

In civil populations of famine-stricken districts it always affects the poorest of the poor first and in greatest numbers. Men between the ages of 40 and 50 develop the disease most readily, women and children less readily, and then only in such households as live in the direst poverty. The idea that certain foodstuffs evoke the disease is disproved. The rapidity with which the œdema can disappear if the diet is improved suggests that if toxic elements play any part in its causation they cannot be very potent, nor their effects very long-lasting. It is more probable that some nutritional defect alters the permeability of the capillary walls. Recent investigations tend to the view that in all forms of œdema some local damage to the capillary endothelium exists. So far as famine-dropsy is concerned no histological evidence has been obtained of damage to the capillary walls. It has been suggested that the deficiency of lipoids in the blood may affect the permeability of the vessel walls. For this lipoid deficiency the absence of fats from the food must bear the principal blame, but it does not follow that a mere absence of fat from the food is the sole cause of the œdema.

Cornish in his observations on prison dietaries and Indian famines persistently attributed the appearance of dropsy to inadequate nitrogenous rather than non-nitrogenous food. Denton and Kohman[6] have stated that dropsy occurs in rats fed on a carrot diet, when the proportion of nitrogen is reduced by the addition of some non-nitrogenous food stuff, such as fat or starch. Kohman[7] found, moreover, that the mere addition of fats, or fat-soluble A, or increase in the salt-content of the diet had no noticeable effect on the occurrence of œdema. But there was more marked œdema when there was much water in the diet than when the animals were on a dry diet. Maver[8] has confirmed these observations, and concludes that the disease is not a specific "vitamine-deficiency" disease, but is in a broader sense a "food-deficiency" disease, resulting from a protracted existence on a diet poor in total calories, and especially poor in protein.

Apart from toxic or nutritional damage to the capillary walls, which may be the result of prolonged under-feeding, there are other possible factors to be considered.

Firstly, there is the altered composition of the blood, which exhibits hydræmia, hypo-albuminosis and deficiency of lipoids. There may be in consequence an increased passage of fluid through the capillary wall. But the blood changes appear to be of too slight a degree; moreover, this theory is not compatible with the rapid resorption of the œdema.

Secondly, there is the hypothesis that the tissue cells, being damaged by nutritional defects, become capable of excessive imbibition of water (Fischer's theory of œdema). There is no evidence in support of this theory, while the fact that the fluid gravitates from one part to another, and can sometimes be drained off by hollow needles, proves that cell-imbibition is not the essential factor.

Thirdly, there is the theory that the endocrine glands (thyroid or adrenals) control the water economy. Several observers are engaged in investigating this theory at the present time, but little more can be said than that they are so engaged.[9] The influence of the endocrine glands upon sugar metabolism encourages the conjecture that some similar influence may be discovered in connection with the water economy.

Other causal factors have been suggested which may be briefly considered.

Can the mere excess of fluid in the diet account for the dropsy? Undoubtedly it cannot. Life can be supported without the appearance of dropsy on a fluid diet provided there is a sufficiency of protein, calories, and vitamines. In the polydipsia and polyuria of diabetes mellitus and insipidus the mere ingestion of fluid does not produce dropsy.

Is the cause to be found in an excessive intake of sodium chloride? Although an excess of sodium chloride may have been a contributory factor amongst prisoners of war, Harden and Zilva's experiments have proved that it is not essential to the production of famine-dropsy.[10]

*Morbid Anatomy.*—The post-mortem appearances which were first observed and recorded by Porter in the Indian famine of 1877–8 are striking and relatively simple.[11] There is a total absence of fat throughout the whole body. At the normal sites for fat deposits, in the subcutaneous tissues, in the omentum and mesentery, about the kidneys and on the heart, fat is replaced by œdema, giving rise to a translucent gelatinous tissue. Hydropericardium, hydrothorax, and ascites are frequently present.

The heart is in all cases greatly atrophied ; the muscle is pale and flabby, exhibiting histologically the changes of brown atrophy. Hypertrophy and dilatation do not occur, the valves remain healthy and competent. The lungs appear small, retracted, anæmic and soft, excepting when broncho-pneumonia and pulmonary œdema occur. The liver is, as a rule, small and, like the kidneys and spleen, pale and soft. Fat and glycogen are absent from the liver cells. The kidneys are healthy, with intact epithelium and tubules. Histologically not only is fat absent from the tissues of all organs and muscles, but the muscle fibres, like the liver cells, are totally devoid of glycogen.

Evidences of concurrent or intercurrent disease are frequently met with, particularly tuberculosis and inflammatory or ulcerative affections of the large intestine. So commonly was some colitis found, that many observers suspected that some specific infection was the cause of famine-dropsy, or that mechanical irritation of the alimentary tract played a part. Maase and Zondek record, however, a group of cases of famine-dropsy without diarrhœa. There is evidence also that the disease is not communicable by contagion.

*Diagnosis.*—Briefly, the diagnosis rests upon a syndrome of four cardinal symptoms : œdema, polyuria, bradycardia, asthenia. There is a noteworthy absence of certain symptoms which frequently accompany other forms of dropsy : cyanosis, dyspnœa, cardiac dilatation, albuminuria.

Unless famine-dropsy is complicated (as often happens)—

by beriberi, no nerve changes will be found; by scurvy, no scorbutic changes (spongy gums, hemorrhages) will be found; by pellagra, the characteristic skin lesions and mental changes seen in pellagrins will be absent.

In the present state of our knowledge it is impossible to say whether the so-called "inanition dropsies" seen in such diseases as phthisis, dysentery, and cancer are or are not identical with famine-dropsy.

Ankylostomiasis presents great difficulty in differential diagnosis, but the presence of profound anæmia with considerable eosinophilia will suggest the necessity for careful examination of the fæces for occult blood and the hook-worm ova.

*Prognosis.*—The prognosis in uncomplicated famine-dropsy is good provided that treatment, in the form of improved diet, can be given. The prospect of rapid and complete recovery is best in young subjects, in those who come earliest under treatment, and in those who are free from any other disease. Old people, infants, and tuberculous subjects fare worst. The mortality is greater in men than in women. The death-rate in persons over 40 years of age has been estimated at 18 per cent., in children between three and four years old at from 22 to 33 per cent.

Von Jaksch's figures deal with the largest total collected by any observer.[12] He records 1,028 deaths out of 22,842 cases, a mortality rate of 4.5 per cent.

Uncomplicated cases begin to recover within 48 hours if kept warm in bed with an increased diet; but relapses are common when the patient first gets up and exerts himself. It may be some weeks before a patient can get up without some slight return of the œdema.

*Treatment.*—The cure of famine-dropsy is simple. Rest in bed, warmth, and a diet rich in carbohydrates appears to cure most cases in a short time. Emphasis must be laid, however, on the latest researches, which prove that an adequate protein content is no less essential to the dietary than an adequate supply of calories.

The diet at first must be light, easily digested, and given in small quantities as in any case of starvation. The amount of liquid and of common salt should be limited. Attention must be paid to any other disease that may be present. Relapses of diarrhœa are in particular apt to be troublesome. The simplest and most compact remedy for famine-dropsy has been found to be cod liver oil.

The prevention of the disease is solely a matter of adequate diet.

*Conclusions.*

1. Famine-dropsy (also called Hunger Œdema and War Œdema) is a form of dropsy associated with bradycardia, polyuria and asthenia.

2. It occurs as the result of prolonged under-feeding, and its appearance is accelerated by physical exertion and exposure to cold.

3. It is a condition independent of renal or cardiac disease, also of beriberi, scurvy, or pellagra.

4. The evidence at present available points to the conclusion that the deficiency is not that of a single food factor, but rather to a combined deficiency in protein and in calories. There is a possibility, moreover, that an excessive intake of fluid in the diet is essential to the production of œdema.

REFERENCES.

[1] Hecker, *Epidemics of the Middle Ages.* London: Sydenham Soc., 1846, p. 231.

[2] Cornish, W., "Observations on the nature of the food of the inhabitants of Southern India, and on prison dietaries in the Madras Presidency," *Madras Quart. Journ. of Med. Science*, 1865, viii.

[3] *Idem.*, "A reply to Sir Richard Temple's minutes of the 7th and 14th March as to the sufficiency of a pound of grain as the basis of famine wages," Madras Government Minute, 1877.

[4] Fontan, "Epidémie d'anasarque essentielle," *Gaz. des Hôp.*, 1919, xcii. 58.

[5] Edgeworth, "On the occurrence of general subcutaneous non-renal œdema as a familial affection," *Lancet*, 1919, ii. 216.

[6] Denton and Kohman, "Feeding experiments with raw and boiled carrots," *J. Biol. Chem.*, 1918, xxxvi. 249.

[7] Kohman, quoted by Maver, *vide infra.*

[8] Maver, "Nutritional edema and war dropsy," *J. Am. M. Ass.*, 1920, lxxiv. 934.

[9] McCarrison, "Dietetic deficiency and endocrine activity, with special reference to deficiency œdemas," *Brit. M. J.*, 1920, i. 236.

[10] Harden and Zilva, "Œdema observed in a monkey fed on a diet free from the fat soluble accessory food factor and low in fat," *Lancet*, 1919, ii. 780.

[11] Porter, *The Diseases of the Madras Famine of* 1877–78. Madras Govt. Press, 1889.

[12] Von Jaksch, "Das Hungerödem," *Wien. klin. Wchnschr.*, 1918, lxviii. 1029.

---

Maase and Zondek, *Das Hungerödem.* Leipzig: Georg Thieme, 1920. (With good bibliography.)

Park, F. S., "War Œdema," *J. Am. M. Ass.*, 1918, lxx. 1826.

Bigland, "Œdema as a symptom in so-called deficiency diseases," *Lancet*, 1920, i. 243.

Enright, "War Œdema in Turkish prisoners of war," *Lancet*, 1920, i. 314.

Nixon, "War Œdema" in *Official Medical History of the War* (in the press).

